# *Clostridioides difficile* colonization among very young children in resource-limited settings

**DOI:** 10.1016/j.cmi.2022.01.022

**Published:** 2022-07

**Authors:** Stephanie A. Brennhofer, Elizabeth T. Rogawski McQuade, Jie Liu, Richard L. Guerrant, James A. Platts-Mills, Cirle A. Warren

**Affiliations:** 1) Division of Infectious Diseases and International Health, School of Medicine, University of Virginia, Charlottesville, VA, USA; 2) Department of Epidemiology, School of Public Health, Emory University, Atlanta, GA, USA; 3) School of Public Health, Qingdao University, Qingdao, China

**Keywords:** *C. difficile*, Children, *Clostridioides difficile*, Diarrhoea, Enteropathy, Low-resource settings, CDI, *C. difficile* infection, *C. difficile*, *Clostridioides difficile*, MPO, myeloperoxidase, NEO, neopterin, AAT, α-1-antitrypsin, AGP, α-1-acid glycoprotein, LMZ, lactulose/mannitol excretion ratio

## Abstract

**Objectives:**

To describe the epidemiology and risk factors for *Clostridioides difficile* (*C. difficile*) colonization among young children in eight low-resource settings.

**Methods:**

We tested 41 354 monthly non-diarrhoeal and diarrhoeal stools for *C. difficile* toxin genes (TcdA and TcdB) using quantitative PCR (qPCR) in 1715 children from birth to age two years in a multisite birth cohort study. We estimated the prevalence, cumulative incidence, and seasonality of *C. difficile* colonization and investigated the associations of *C. difficile* detection with risk factors of infection, markers of enteropathy, and growth.

**Results:**

The prevalence of *C. difficile* detection was lower in diarrhoeal (2.2%; *n* = 151/6731) compared to non-diarrhoeal stools (6.1%; *n* = 2106/34 623). By 24 months of age, the cumulative incidence of *C. difficile* varied widely by site, with 17.9% (*n* = 44; Pakistan) to 76.3% (*n* = 148; Peru) of children having at least one positive stool. Only Bangladesh and Pakistan had seasonal differences in *C. difficile* detection. Female sex (adjusted risk ratio (aRR): 1.18; 95% CI: 1.02–1.35), cephalosporin use in the past 15 days (aRR: 1.73; 95% CI: 1.39–2.16), and treated water (aRR: 1.24; 95% CI: 1.02–1.50) were risk factors for *C. difficile* positivity. The presence of *C. difficile* was significantly associated with elevated faecal myeloperoxidase, neopterin, and α-1-antitrypsin, but no associations were found between *C. difficile* and child growth at 24 months of age.

**Discussion:**

*C. difficile* colonization among children ages 0–2 years was variable across low-resource settings. Significant elevation of intestinal inflammation and barrier disruption markers associated with *C. difficile* detection suggests a subclinical impact of colonization.

## Introduction

High rates of *Clostridioides difficile* colonization have been documented among infants in resource-sufficient settings, where *C. difficile* infection (CDI) rates can be as high as 90% among hospitalized neonates [1]. Colonization decreases with increasing age until age two, when rates start to mirror those in healthy adults. Routine testing for *C. difficile* is not recommended for patients younger than two years of age [2]. The prevalence of *C. difficile* colonization and infection among children in low-resource settings is difficult to ascertain and likely underestimated given a lack of awareness and availability of testing [3]. An earlier study from Nigeria reported a 14.8% overall frequency of toxigenic *C. difficile* detection among neonates and children [4]. In Brazil, colonization rates ranged from 0% to 25% in stool from healthy children [5]. Some studies have indicated that *C. difficile* may be more prevalent than rotavirus or *Cryptosporidium* in children presenting with diarrhoea to the hospital [6].

This study aimed to describe the epidemiology and risk factors for *C. difficile* colonization among children up to two years of age in the Etiology, Risk Factors, and Interactions of Enteric Infections and Malnutrition and the Consequences for Child Health and Development (MAL-ED) multi-site birth cohort study. Furthermore, we investigated whether *C. difficile* detection contributed to longer-term health outcomes, including markers of environmental enteropathy and growth shortfalls.

## Methods

### Study design, settings, and participants

The MAL-ED study design and methodology have been previously described [7]. The study was conducted at eight sites: Dhaka, Bangladesh; Fortaleza, Brazil; Vellore, India; Bhaktapur, Nepal; Loreto, Peru; Naushero Feroze, Pakistan; Venda, South Africa; and Haydom, Tanzania. The study ran from November 2009 through February 2014. Children were enrolled from birth (<17 days of age) and followed until 24 months of age with twice-weekly home visits. Of the 2145 children enrolled in the MAL-ED study, there was complete two-year follow-up data for 1715 children (80%) [8]. Of the 44 570 stools collected, 42 630 (95.6%) had sufficient specimen available and 41 354 (92.4%) had valid quantitative PCR (qPCR) results for toxigenic *C. difficile* to be included in this analysis. Surveillance stool samples (in the absence of diarrhoea) were collected monthly. In case of diarrhoea, stool samples were collected from each diarrhoeal episode. A biannual survey, starting at six months of age, collected sociodemographic information such as household income, maternal education, source and treatment of water, sanitation, and ownership of animals. Water was considered to be treated if filtered or boiled or if bleach was added. Water was considered to be from an improved source if the source was constructed to deliver safe drinking water, and sanitation facilities were considered improved if they successfully kept human excrement away from human contact [9]. Weight-for-age and length-for-age z-scores were calculated monthly [10].

### Microbiology

The QIAmp Fast DNA Stool Mini Kit (Qiagen) was used to extract total nucleic acid from the stool specimens [8]. TaqMan Array Cards were developed to detect 29 enteropathogens via qPCR using the AgPath One Step real-time PCR kit (Thermo-Fisher), as previously detailed [8]. We specifically studied toxigenic *C. difficile*, and all mentions of *C. difficile* in the following text refer to toxigenic *C. difficile*. The qPCR assays targeting *C. difficile* TcdA [11] and TcdB [12] genes were validated on the TaqMan Array platform, demonstrating 100% sensitivity and 100% specificity on clinical specimens using a secondary real-time PCR as confirmation [13]. The quantification cycle for positivity by both assays was set at <35 [8]. Monthly stool samples were tested for myeloperoxidase (MPO; measured in ng/mL), neopterin (NEO; measured in nmol/L), and α-1-antitrypsin (AAT; measured in mg/g) and analyzed on the logarithmic scale. Serum α-1-acid glycoprotein (AGP; measured in mg/dL) was measured at months 7, 15, and 24. Lactulose/mannitol excretion ratios were measured in urine at 3, 6, 9, and 15 months and converted to sample-based z-scores (LMZ), as described previously [14]. Plasma zinc and retinol were measured at 7, 15, and 24 months.

### Statistical methods

To determine whether *C. difficile* was a risk factor for diarrhoeal vs. surveillance stools, we ran a Poisson regression to estimate risk ratios (RRs) with generalized estimating equations (GEE) to account for repeated measures. Because *C. difficile* was not associated with diarrhoea, which is consistent with previous analyses of the MAL-ED cohort [8], only surveillance stools were included in the rest of the analyses. The cumulative incidence and prevalence of non-diarrhoeal *C. difficile* detection was calculated by site. Seasonality of *C. difficile* colonization was described by site by modelling *C. difficile* detection with a linear term for age in days and the terms sin (2π*w*/(52)) + cos (2π*w*/52) + sin (4π*w*/52) + cos (4π*w*/52), where *w* denotes the week of the year [15]. To determine whether seasonal variation was statistically significant for any one site, a reduced model without the sine and cosine terms was compared to the full model via an analysis of variance (ANOVA) for each site. Associations between putative risk factors and *C. difficile* detection in monthly surveillance stool samples were estimated using log-binomial regression to estimate RR with GEE.

The association between *C. difficile* and intestinal permeability and inflammatory markers was evaluated in concurrent monthly surveillance stool samples using linear regression with GEE. The associations between *C. difficile* burden and anthropometry at 24 months of age were estimated using linear regression models. *C. difficile* burden was summarized as the proportion of surveillance stool samples positive for *C. difficile* from 1 to 24 months of age. Effects were categorized as no burden (0%), low burden (1%–9%), and high burden (≥10%). All statistical analyses were performed via R software, version 4.0.2 (Foundation for Statistical Computing).

### Ethics

We obtained ethical approval from the institutional review boards at each of the participating research sites, at the Johns Hopkins Bloomberg School of Public Health (Baltimore, MD, USA) and at the University of Virginia School of Medicine (Charlottesville, VA, USA). Additionally, all sites received ethical approval from their respective governmental, local institutional, and collaborating institutional review boards. Written informed consent was obtained from the parent or guardian of each child.

## Results

We included 34 623 non-diarrhoeal monthly surveillance and 6731 diarrhoeal stool samples that were tested for *C. difficile* from 1715 children over a two-year period of time (Fig. S1). *C. difficile* was detected in 2106 of non-diarrhoeal stool samples (6.1%) from 832 children (48.5%), of whom 464 had recurrent detection in subsequent surveillance (Table 1). Included children were similar to those who did not complete follow-up by most baseline sociodemographic characteristics (Table S1). *C. difficile* detection was associated with a higher detection of astrovirus and typical enteropathogenic *Escherichia coli* and a lower detection of *Cryptosporidium*, *Enterocytozoon bieneusi*, and *Giardia*, after adjusting for age (Table S2).

**Table 1:** Characteristics among the 1715 children enrolled in the MAL-ED birth cohort

Characteristics	Bangladesh (*n* = 210)	Brazil (*n* = 165)	India (*n* = 227)	Nepal (*n* = 227)	Peru (*n* = 194)	Pakistan (*n* = 246)	South Africa (*n* = 237)	Tanzania (*n* = 209)	All (*n* = 1715)
Child characteristics									
Female, *n* (%)	102 (48.6)	76 (46.1)	122 (53.7)	105 (46.3)	89 (45.9)	126 (51.2)	117 (49.4)	104 (49.8)	841 (49.0)
Enrolment WAZ, mean ± SD^a^	–1.26 ± 0.94	–0.16 ± 1.05	–1.30 ± 1.04	–0.92 ± 0.97	–0.62 ± 0.91	..	–0.38 ± 0.95	–0.13 ± 0.94	–0.70 ± 1.07
Enrolment LAZ, mean ± SD^a^	–0.97 ± 1.01	–0.80 ± 1.13	–1.02 ± 1.05	–0.72 ± 1.03	–0.95 ± 0.96	..	–0.71 ± 1.00	–1.03 ± 1.14	–0.88 ± 1.05
24-mo WAZ, mean ± SD^a^	–1.61 ± 0.99	0.39 ± 1.21	–1.65 ± 0.94	–0.93 ± 0.90	–0.79 ± 0.90	..	–0.51 ± 0.98	–1.33 ± 1.01	–0.96 ± 1.17
24-mo LAZ, mean ± SD^a^	–2.03 ± 0.94	–0.04 ± 1.08	–1.92 ± 0.97	–1.35 ± 0.92	–1.88 ± 0.87	..	–1.70 ± 1.06	–2.67 ± 1.02	–1.70 ± 1.20
Days exclusively breastfed, mean ± SD^b^	143.20 ± 42.74	93.74 ± 57.77	105.44 ± 42.92	92.47 ± 54.46	89.48 ± 61.35	19.88 ± 22.69	38.56 ± 26.26	62.15 ± 34.97	78.63 ± 57.74
Vitamin A deficiency at 7 mo, *n* (%)^c^	96 (52.2)	22 (18.3)	40 (19.7)	63 (29.4)	61 (35.1)	120 (53.1)	126 (84.0)	88 (87.1)	616 (44.9)
Zinc deficiency at 7 mo, *n* (%)^d^	43 (23.5)	5 (4.2)	110 (50.9)	66 (30.8)	3 (1.7)	182 (78.8)	..	27 (24.8)	436 (35.0)
At least one *C. difficile–*positive stool, *n* (%)	85 (40.5)	103 (62.4)	72 (31.7)	109 (48.0)	148 (76.3)	44 (17.9)	160 (67.5)	111 (53.1)	832 (48.5)
Sociodemographics									
WAMI score, mean ± SD	0.55 ± 0.13	0.84 ± 0.08	0.49 ± 0.15	0.70 ± 0.13	0.55 ± 0.12	0.49 ± 0.19	0.78 ± 0.11	0.22 ± 0.12	0.57 ± 0.22
Household income (≥150 USD), *n* (%)	69 (32.9)	161 (97.6)	19 (8.4)	106 (46.7)	58 (29.9)	115 (46.7)	179 (75.5)	0 (0.0)	707 (41.2)
Maternal education (≥6 y), *n* (%)^e^	144 (68.6)	164 (99.4)	193 (85.0)	204 (89.9)	188 (96.9)	108 (43.9)	237 (100.0)	160 (76.6)	1398 (81.5)
Maternal age, mean ± SD^f^	25.04 ± 5.03	25.38 ± 5.56	23.87 ± 4.18	26.63 ± 3.70	24.79 ± 6.25	28.07 ± 5.92	27.04 ± 7.22	29.10 ± 6.54	26.31 ± 5.89
Water and sanitation									
Improved source of drinking water, *n* (%)	210 (100.0)	165 (100.0)	227 (100.0)	227 (100.0)	184 (94.8)	246 (100.0)	196 (82.7)	89 (42.6)	1544 (90.0)
Treated water, *n* (%)	130 (61.9)	10 (6.1)	7 (3.1)	98 (43.2)	32 (16.5)	0 (0.0)	12 (5.1)	12 (5.7)	301 (17.6)
Access to improved latrine, *n* (%)	210 (100.0)	165 (100.0)	121 (53.3)	227 (100.0)	66 (34.0)	197 (80.1)	232 (97.9)	19 (9.1)	1237 (72.1)
Environment									
Owned chickens, *n* (%)	3 (1.4)	0 (0.0)	10 (4.4)	60 (26.4)	51 (26.3)	94 (38.2)	71 (30.0)	194 (91.9)	481 (28.0)
Owned cattle, *n* (%)	1 (0.5)	0 (0.0)	5 (2.2)	3 (1.3)	0 (0.0)	146 (59.3)	33 (13.9)	157 (75.1)	345 (20.1)
Dirt floor, *n* (%)	6 (2.9)	0 (0.0)	5 (2.2)	118 (52.0)	125 (64.4)	165 (67.1)	6 (2.5)	196 (93.8)	621 (36.2)

LAZ, length for age z score; SD, standard deviation; USD, United States dollar; WAMI, water, assets, maternal education, income; WAZ, weight for age z score.

a Pakistan excluded (*n* = 246).

b Days of exclusive breastfeeding includes all days (not just the first 6 months).

c Missing data (*n* = 343).

d South Africa excluded (*n* = 237). Additional missing data (*n* = 232).

e Missing data (*n* = 2) were imputed based on country average and then rounded to the nearest whole number.

f Missing data (*n* = 2) were based on site mean.

### Prevalence and cumulative incidence

**Fig. 1:**
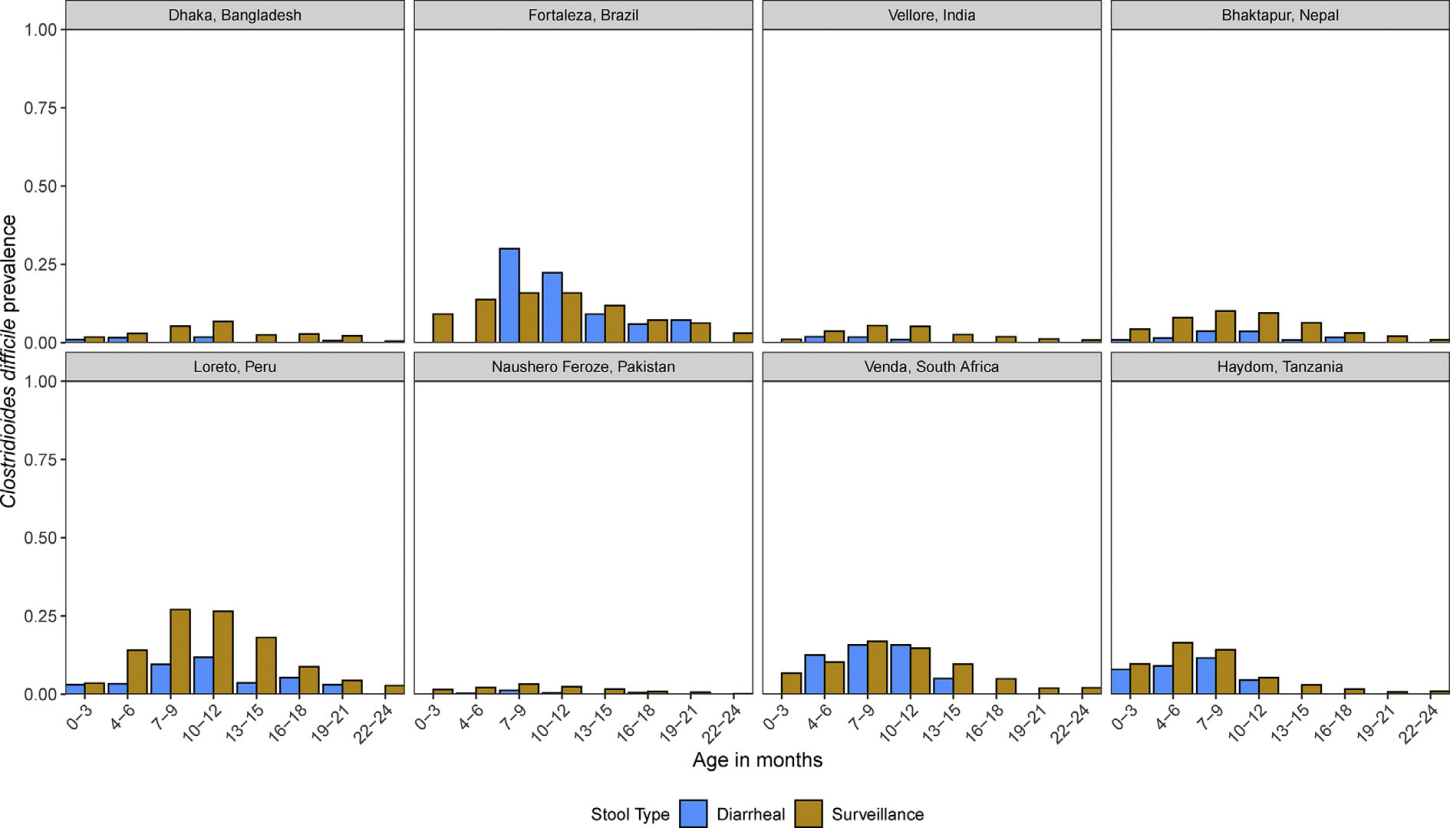
*Clostridioides difficile* prevalence in diarrhoeal and surveillance stool samples by age in months among 1715 children enrolled in the Etiology, Risk Factors, and Interactions of Enteric Infections and Malnutrition and the Consequences for Child Health and Development birth cohort.

**Fig. 2:**
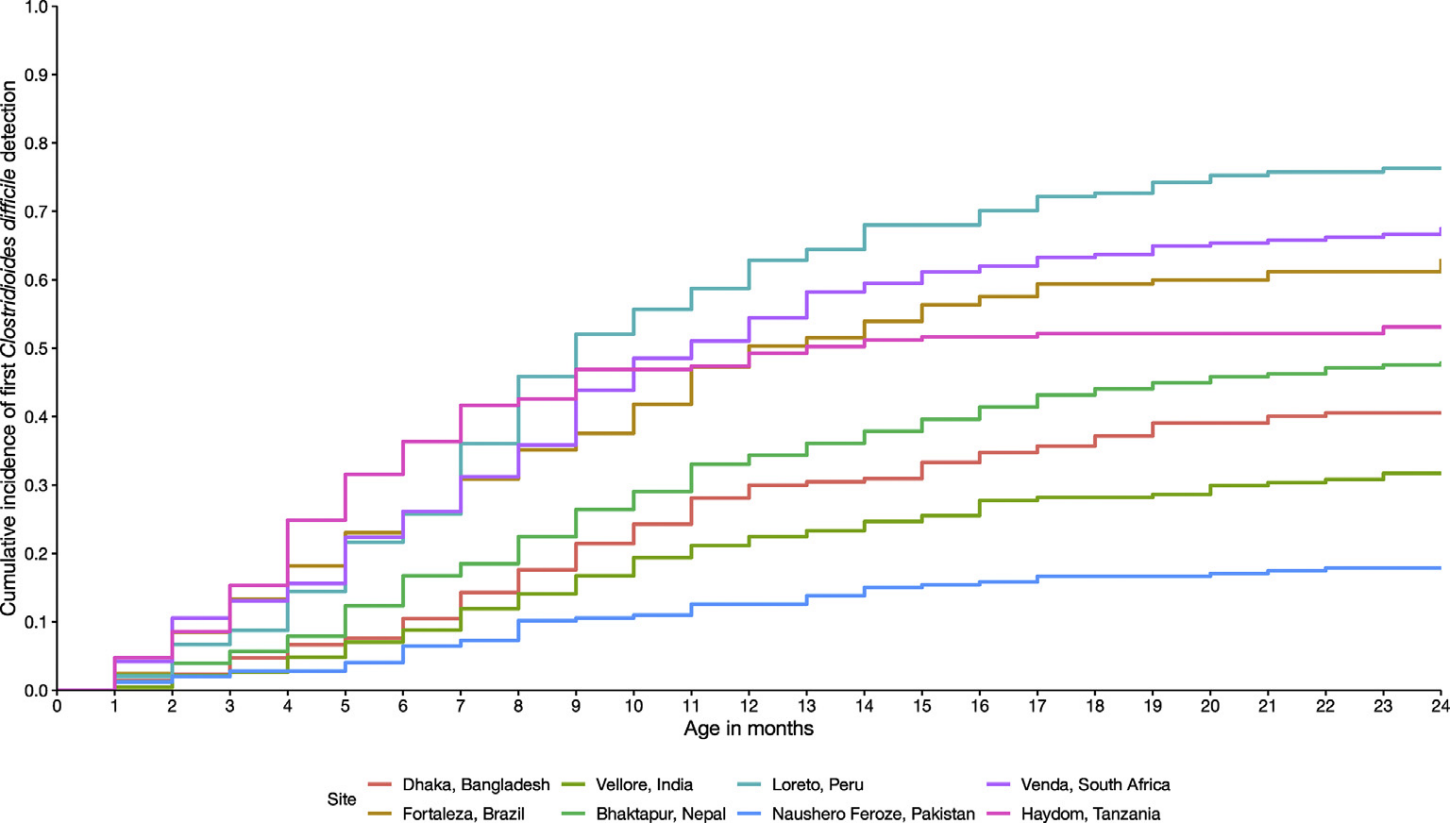
Cumulative incidence of *Clostridioides difficile* detection in surveillance stool samples among 1715 children enrolled in the Etiology, Risk Factors, and Interactions of Enteric Infections and Malnutrition and the Consequences for Child Health and Development birth cohort.

The prevalence of *C. difficile* was lower in diarrhoeal stools compared to surveillance stools (p < 0.001) (Fig. 1). *C. difficile* was most prevalent during the first year of life and declined during the second year of life (p < 0.001). Peru had the highest prevalence of *C. difficile* detection in surveillance stools, which peaked from 7–12 months of age. In contrast, Pakistan had the lowest prevalence of *C. difficile* detection in surveillance stool samples, with the highest prevalence of only 3.2% at ages 7–9 months. Peru had the highest and Pakistan had the lowest cumulative incidence of *C. difficile* detection: 76.3% and 17.9% of all children had at least one stool positive for *C. difficile* by age 24 months, respectively (Fig. 2).

### Seasonality

The prevalence of *C. difficile* was relatively consistent throughout the year in most sites except for Bangladesh and Pakistan (Fig. S2), where seasonal differences were statistically significant. In Bangladesh, the peaks for *C. difficile* detection were observed during the rainy season (June–October). Although prevalence was low in Pakistan year-round, increased rates were noted during the dry season (March–May).

### Risk factors

In this cohort, we found that recent antibiotic use had variable associations with *C. difficile* detection (Table 2)*.* Cephalosporin use in the 0–15 days prior to surveillance stool collection was a risk factor for *C. difficile* detection (aRR: 1.73; 95% CI: 1.39–2.16) and when adjusted for diarrhoea 0–15 days prior to stool collection, the estimate grew stronger (aRR: 1.81; 95% CI: 1.45–2.25). Macrolide use 0–15 days and 16–30 days prior to stool collection was associated with a 34% (aRR: 0.66; 95% CI: 0.51–0.84) and 31% (aRR: 0.69; 95% CI: 0.55–0.88) decreased risk of *C. difficile* detection, respectively. Recent diarrhoea (0–15 days prior to the surveillance stool collection) was associated with lower rates of subsequent *C. difficile* detection (aRR: 0.71; 95% CI: 0.60–0.83); the estimate did not change when adjusted for macrolide and cephalosporin use. There was no association between recent hospitalization or presentation to clinic and *C. difficile* detection. Remarkably, treated water was associated with an increased risk of *C. difficile* detection, even when adjusted for socioeconomic status. On further investigation, this effect was largely driven by the Bangladesh (aRR: 3.45; 95% CI: 2.05–5.78) and Nepal (aRR: 1.66; 95% CI: 1.11–2.49) sites. Among infants aged 0–6 months, proportion of days exclusively breastfed in the last month was marginally associated with decreased *C. difficile* detection (aRR: 0.76; 95% CI: 0.57–1.00).

**Table 2:** Risk factors for *C. difficile* detection in 34 623 monthly surveillance stool samples among 1715 children enrolled in the MAL-ED birth cohort

Risk factors	*n* (%)	Unadjusted risk ratios (95% CI)	Adjusted risk ratios (95% CI)^a^
Child characteristics			
Female sex	17 034 (49.2)	1.11 (0.96–1.28)	1.18 (1.02–1.35)
Antibiotic exposure			
Any antibiotic (past 0–15 d)^b^	7394 (21.4)	1.02 (0.92–1.12)	1.03 (0.93–1.14)
Any antibiotic (past 16–30 d)^b^	7140 (20.6)	1.01 (0.91–1.11)	1.01 (0.91–1.12)
Cephalosporins (past 0–15 d)	1816 (5.3)	1.19 (1.03–1.37)	1.73 (1.39–2.16)
Cephalosporins (past 16–30 d)	1735 (5.0)	0.91 (0.77–1.08)	1.23 (0.94–1.60)
Macrolides (past 0–15 d)	1434 (4.1)	0.72 (0.57–0.91)	0.66 (0.51–0.84)
Macrolides (past 16–30 d)	1345 (3.9)	0.77 (0.62–0.97)	0.69 (0.55–0.88)
Penicillin (past 0–15 d)	3414 (9.9)	1.11 (0.98–1.27)	1.07 (0.94–1.22)
Penicillin (past 16–30 d)	3186 (9.2)	1.15 (1.00–1.31)	1.06 (0.92–1.22)
Sulfonamides (past 0–15 d)	769 (2.2)	1.00 (0.76–1.31)	0.92 (0.70–1.20)
Sulfonamides (past 16–30 d)	711 (2.1)	1.14 (0.89–1.47)	1.09 (0.85–1.40)
Fluoroquinolones (past 0–15 d)	315 (0.9)	0.58 (0.35–0.98)	0.70 (0.33–1.49)
Fluoroquinolones (past 16–30 d)	299 (0.9)	0.98 (0.64–1.49)	1.33 (0.78–2.27)
Metronidazole (past 0–15 d)	834 (2.4)	0.68 (0.52–0.88)	0.75 (0.51–1.09)
Metronidazole (past 16–30 d)	987 (2.9)	0.95 (0.76–1.18)	1.09 (0.81–1.46)
Potential healthcare exposure			
Diarrhoea prior to stool collection (past 0–15 d)	3227 (9.3)	0.77 (0.65–0.90)	0.71 (0.60–0.83)
Diarrhoea prior to stool collection (past 16–30 d)	3964 (11.4)	1.08 (0.95–1.21)	0.97 (0.85–1.10)
Hospitalized prior to stool collection (past 0–15 d)	270 (0.8)	1.22 (0.80–1.86)	1.14 (0.74–1.75)
Hospitalized prior to stool collection (past 16–30 d)	288 (0.8)	1.00 (0.64–1.54)	0.91 (0.58–1.43)
Presented to clinic (past 0–15 d)	4131 (11.9)	1.02 (0.91–1.15)	0.93 (0.82–1.04)
Presented to clinic (past 16–30 d)	4077 (11.8)	1.06 (0.93–1.20)	0.91 (0.81–1.03)
Sociodemographics			
Income (≥150 USD)	13 919 (40.2)	1.30 (1.12–1.50)	1.03 (0.86–1.24)
Maternal education (≥6 y)	28 152 (81.3)	2.07 (1.63–2.64)	0.94 (0.73–1.20)
Maternal age (per 5-y increase in age)	—	0.98 (0.92–1.05)	1.01 (0.95–1.06)
Water and sanitation			
Improved source of drinking water	31 173 (90.0)	0.88 (0.71–1.08)	1.13 (0.89–1.44)
Treated water	6390 (18.5)	1.06 (0.89–1.26)	1.24 (1.02–1.50)
Access to improved latrine	24 738 (71.4)	0.90 (0.76–1.05)	1.18 (0.94–1.49)
Environment			
Owned chickens	12 249 (35.4)	0.92 (0.80–1.07)	0.89 (0.74–1.07)
Owned cattle	6834 (19.7)	0.60 (0.49–0.73)	0.82 (0.64–1.05)
Dirt floor	12 760 (36.9)	0.95 (0.82–1.11)	0.88 (0.71–1.09)

CI, confidence interval; USD, United States dollar.

a Adjusted for site and age (with a natural spline and 3 degrees of freedom).

b Cephalosporins, macrolides, penicillin, sulfonamides, fluoroquinolones, metronidazole, and tetracycline.

### Associations with intestinal permeability and inflammatory markers

Overall, *C. difficile* detection was associated with elevated MPO (mean difference: 0.12 log (ng/mL); 95% CI: 0.05–0.19), NEO (mean difference: 0.18 log (nmol/L); 95% CI: 0.12–0.23), and AAT (mean difference: 0.20 log (mg/g); 95% CI: 0.15–0.26) but decreased AGP (mean difference: –6.21 mg/dL; 95% CI: –11.61 to –0.82) (Table 3). When the model for *C. difficile* detection and MPO was further adjusted for *Campylobacter* and *Shigella*, two inflammatory pathogens, and another five pathogens (astrovirus, *Cryptosporidium*, *E. bieneusi*, *Giardia*, and typical enteropathogenic *E. coli*) associated with *C. difficile*, the estimates did not change. The estimates for NEO, AAT, AGP, and LMZ also did not change when further adjusted for the five pathogens associated with *C. difficile* detection.

**Table 3:** Associations between *C. difficile* detection and markers of inflammation and gut permeability in monthly surveillance stool samples among 1715 children enrolled in the MAL-ED birth cohort

Site	MPO difference^a^*n* = 18 851	NEO difference^a^*n* = 18 851	AAT difference^a^*n* = 18 851	AGP difference^a^*n* = 3586	LMZ difference^a^*n* = 4934
Bangladesh	0.20 (–0.04 to 0.44)	0.17 (–0.06 to 0.40)	0.16 (–0.03 to 0.35)	–16.27 (–28.22 to –4.33)	–0.24 (–0.66 to 0.18)
Brazil	0.04 (–0.20 to 0.28)	0.02 (–0.16 to 0.20)	0.17 (0.03–0.31)	2.67 (–9.59 to 14.93)	–0.02 (–0.25 to 0.21)
India	0.21 (0.01–0.42)	0.35 (0.15–0.56)	0.29 (0.08–0.51)	–21.81 (–35.97 to –7.64)	–0.27 (–0.59 to 0.05)
Nepal	0.15 (0.00–0.30)	0.11 (0.03–0.20)	0.03 (–0.12 to 0.19)	–10.84 (–22.29 to 0.61)	–0.01 (–0.25 to 0.24)
Peru	0.19 (0.03–0.35)	0.28 (0.18–0.38)	0.43 (0.33–0.53)	–3.91 (–15.52 to 7.70)	–0.04 (–0.17 to 0.10)
Pakistan	–0.28 (–0.59 to 0.04)	0.00 (–0.23 to 0.23)	0.16 (–0.18 to 0.49)	–15.83 (–40.99 to 9.33)	–0.05 (–0.40 to 0.30)
South Africa	0.06 (–0.06 to 0.18)	0.01 (–0.12 to 0.13)	–0.01 (–0.14 to 0.12)	–3.35 (–17.68 to 10.99)	0.39 (0.11–0.68)
Tanzania	–0.04 (–0.23 to 0.15)	0.25 (0.07–0.44)	0.15 (0.00–0.30)	–9.43 (–27.00 to 8.15)	–0.24 (–0.69 to 0.22)
All^b^	0.12 (0.05–0.19)	0.18 (0.12–0.23)	0.20 (0.15–0.26)	–6.21 (–11.61 to –0.82)	0.01 (–0.10 to 0.11)

AGP, α-1-acid glycoprotein (mg/dL); AAT, α-1-antitrypsin (log(mg/g)); LMZ, lactulose/mannitol z score; MPO, Myeloperoxidase (log(ng/mL)); NEO, Neopterin (log(nmol/L)).

a Adjusted for age.

b Additionally adjusted for site.

### Effects on growth

There were no associations between *C. difficile* and weight or length attainment at 24 months of age. In children with a high *C. difficile* burden (≥10% stool specimen tested were positive), there was a –0.04 z-score difference in weight (95% CI: –0.18 to 0.10) and a 0.01 z-score difference in length (95% CI: –0.12 to 0.14) compared to children with no *C. difficile* infections (Table S3).

## Discussion

We demonstrated that detection of toxigenic *C. difficile* in surveillance stools among children in resource-limited settings is low but varies by site. However, most children were transiently colonized with *C. difficile*, especially during first year of life. Except in Brazil, *C. difficile* was typically isolated from non-diarrhoeal stools, which is consistent with work previously published from this cohort [8]. In contrast, studies from high-income countries indicate that *C. difficile* is an important cause of both community- and hospital-acquired infections [16,17]. Although antibiotic-induced disruption of the gut microbiota is likely common globally, healthcare exposure leading to increased likelihood for acquisition of clostridial spores may be more prevalent in resource-sufficient settings. Thus, the lack of association with clinic or hospital exposure in this cohort may be due to limited access to healthcare and pathogen acquisition elsewhere.

Detection of *C. difficile* in asymptomatic children in the community in low-resource settings suggests potential but undefined environmental sources of *C. difficile*. Indeed, others have reported environmental exposures, including pets, to be associated with a diagnosis of CDI in children [18]. Two sites (Bangladesh and Pakistan) showed contrasting seasonality of *C. difficile* detection, hinting that there may be site-specific climatic conditions that facilitate cyclical exposure to contaminated water, soil, animals, or particular produce or other food items. Interestingly, “treated water,” which could be a surrogate marker of environmentally contaminated water supply, was identified as a risk factor. Consistent with a recent review on asymptomatic *C. difficile* colonization among children [19], we observed a peak in *C. difficile* detection at around 7–12 months, which corresponds to the introduction of complementary foods, indicating environmental exposure as a potential source.

Unregulated dispensing of antibiotics is rampant in resource-limited settings [20]. However, the burden of CDI (or colonization) does not appear to be as elevated in low-resource settings as it is in high-resource countries. Lack of awareness, cost of testing, and empiric treatment (e.g. metronidazole use for diarrhoea), may mask the true prevalence of this antibiotic-associated diarrhoeal disease [3]. Among the antibiotics analyzed in this study, cephalosporins were positively associated with detection of *C. difficile* in surveillance stools. Unexpectedly, macrolide exposure was noted to be protective against *C. difficile* colonization. The most common macrolides used in this cohort were azithromycin and erythromycin [20]. Although beneficial against childhood mortality, especially in the very young [21], azithromycin has been known to decrease gut bacterial composition and diversity [22,23]. Indeed, macrolides have been one of the antibiotic classes identified to be a risk factor for community acquired CDI [24]. However, published findings on the effect of macrolides on microbiota composition and diversity vary depending on study site and host age. Thus, macrolide exposure may not always result in decreased colonization resistance against *C. difficile.* Furthermore, the macrolide azithromycin has activity against *C. difficile* strains, especially in non-BI/NAP1/27 ribotypes [25]. Although there is some evidence that dysbiosis secondary to other diarrheagenic enteric pathogens increases susceptibility to *C. difficile* colonization [26], it is also possible that the presence of other pathogenic flora may present a competitive niche against *C. difficile*, which could explain the protective effect of prior diarrhoea observed in this study. Other potential factors that may have influenced colonization resistance during diarrhoeic episodes may include culture-specific practices such as dietary manipulation [27,28] and use of traditional medicines [29], which were not captured.

Although not a significant cause of diarrhoea and stunting, *C. difficile* was associated with markers of environmental enteropathy [30]. Faecal inflammatory markers, MPO and/or NEO, were elevated in children with *C. difficile* in India, Nepal, Peru, and Tanzania. A marker for protein exudation in the intestinal mucosa, ATT, was seen in children from Brazil, India, Peru, and Tanzania. Overall, these findings suggest that the presence of *C. difficile* may elicit a local host response, but the clinical significance remains to be investigated. These faecal markers of enteropathy have not been studied in children in resource-sufficient settings, although faecal cytokines were not shown to be elevated in asymptomatic CDI in a study from a children's hospital in the United Kingdom [31]. Whether intestinal inflammation from other causes influenced microbiota composition leading to eventual *C. difficile* colonization in children in our study sites is unclear. More studies are warranted to confirm whether intestinal carriage of *C. difficile* indeed has subclinical impact and whether this observation is unique to low-resource settings.

Our study is limited by the reliance on the multiplex-gene assay for detection of *C. difficile* in stool. Although this molecular assay is highly sensitive in determining whether a child has toxigenic *C. difficile* in the stool, lack of actual toxin antigen detection may need to be confirmed to explain the absence of diarrhoea in colonized individuals. On the other hand, the presence of even minute amounts of toxin could potentially illuminate why inflammatory markers were elevated during asymptomatic infection. In addition to toxin detection, culture, susceptibility testing, and strain characterization of the *C. difficile* isolated and microbiota analysis in a similar prospectively followed cohort of children are needed in future studies.

In summary, transient *C. difficile* colonization is prevalent among young children in resource-limited countries. Risk factors for *C. difficile* detection may be distinct in this setting. Although not associated with diarrhoea and growth shortfalls, asymptomatic *C. difficile* infection was associated with significant elevation of markers of intestinal inflammation, suggesting subclinical impact of carriage. These findings imply the need to extend antimicrobial and diagnostic stewardship and infection prevention measures to the community to address the threat of *C. difficile* in children.

## Transparency declaration

The authors report no conflicts of interest. The Etiology, Risk Factors and Interactions of Enteric Infections and Malnutrition and the Consequences for Child Health and Development Project (MAL-ED) is a collaborative project supported by the Bill & Melinda Gates Foundation (OPP1131125), and the Fogarty International Center. We thank the staff and participants of the MAL-ED Network Project for their important contributions. CAW is partially supported by NIH AI145322. The findings and conclusions in this report are those of the authors and do not necessarily represent the official position of the US National Institutes of Health or Department of Health and Human Services.

## Author contributions

Concept and design: CAW; acquisition, analysis, or interpretation of data: SAB, ETRM, JL, JAP-M, CAW; critical revision of the manuscript for important intellectual content: all authors; statistical analysis: SAB, ETRM, JAP-M; administrative, technical, or material support: RLG, CAW; supervision: ETRM, JAP-M, CAW.
