# Supplementary material for: Clostridioides difficile colonization among very young children in resource-limited settings
Source: Clin Microbiol Infect. 2022 Jul;28(7):996–1002. doi: 10.1016/j.cmi.2022.01.022 (PMC9240321; doi:10.1016/j.cmi.2022.01.022)
Supplement: Multimedia component 1 [file mmc1.docx]

**Supplementary Online Content**

*Clostridioides difficile* colonization among very young children in resource-limited settings

Stephanie A. Brennhofer, Elizabeth T. Rogawski McQuade, Jie Liu, Richard L. Guerrant, James A. Platts-Mills, Cirle A. Warren

**eFigure 1.** Flow of participants and samples amongst the children enrolled in the MAL-ED birth cohort.

**eTable 1.** Demographic comparison between the children included and excluded in the statistical analyses amongst the children enrolled in the MAL-ED birth cohort.

**eTable 2.** Associations between *C. difficile* and other enteric pathogens among 1715 children enrolled in the MAL-ED birth cohort.

**eFigure 2.** Prevalence of *Clostridioides difficile* detection in surveillance stools over months of the year among 1715 children enrolled in the MAL-ED birth cohort.

**eTable 3.** Effects of *Clostridioides difficile* burden in monthly surveillance stool samples on weight and length attainment at 2 years of age among 1469 children enrolled in the MAL-ED birth cohort.

eFigure 1. Flow of participants and samples amongst the children enrolled in the MAL-ED birth cohort.

Total number of enrolled children (N=2145)

Toxigenic C*. difficile* detected (N=2106)

Stools with sufficient specimen (N=42630)

Stools with valid qPCR for toxigenic *C. difficile* (N=41354)

Surveillance stools (N=34623)

Diarrhoeal stools

(N=6731)

Toxigenic C*. difficile* detected (N=151)

Total stools collected (N=44570)

Children with 2 years of complete follow-up (N=1715)

**eTable 1.** Demographic comparison between the children included and excluded in the statistical analyses amongst the children enrolled in the MAL-ED birth cohort.

| Characteristics | Excluded children  (N=430)^a^ | Included children (N=1715) |
| --- | --- | --- |
| Child characteristics |  |  |
| Female (n, %) | 234 (54.4) | 841 (49.0) |
| Enrollment WAZ^b^ (mean, SD) | -0.79 ± (1.10) | -0.80 ± (1.09) |
| Enrollment LAZ^b^ (mean, SD) | -1.08 ± (1.17) | -0.95 ± (1.08) |
| Days exclusively breastfed^c^ (mean, SD) | 70.69 ± (69.08) | 78.63 ± (57.74) |
| Sociodemographics |  |  |
| WAMI score^d^ (n, %) | 0.55 (0.21) | 0.57 (0.22) |
| Household income (>150 USD)^e^ (n, %) | 100 (40.5) | 707 (41.2) |
| Maternal education (>6 years)^f^ (n, %) | 175 (70.6) | 1093 (63.8) |
| Maternal age^g^ (mean, SD) | 24.15 ± (5.44) | 26.31 ± (5.90) |
| Water and sanitation |  |  |
| Improved source of drinking water^d^ (n, %) | 212 (85.5) | 1544 (90.0) |
| Treated water^d^ (n, %) | 60 (24.2) | 301 (17.6) |
| Access to improved latrine^d^ (n, %) | 169 (68.1) | 1237 (72.1) |
| LAZ = length for age z score. SD = standard deviation. USD = United States dollar. WAMI = Water, Assets, Maternal Education, Income. WAZ = weight for age z score. ^a^ Children were excluded due to a lack of two-year follow-up data. ^b^ Pakistan excluded (N=246). ^c^ Days of exclusive breastfeeding includes all days (not just the first 6 months). Missing data (N=12/430). ^d^ Missing data (N=182/430). ^e^ Missing data (N=183/430). ^f^ Missing data (N=182/430; N=2/1715). ^g^ Missing data (N=2/1715). | | |

**eTable 2.** Associations between *C. difficile* and other enteric pathogens among 1715 children enrolled in the MAL-ED birth cohort.

| Pathogen | Risk Ratio (95% CI) |
| --- | --- |
| Adenovirus 40/41 | 1.04 (0.91, 1.20) |
| Astrovirus | 1.16 (1.01, 1.34) |
| *Campylobacter* | 1.05 (0.93, 1.18) |
| *Cryptosporidium* | 0.71 (0.56, 0.90) |
| Norovirus | 0.99 (0.87, 1.12) |
| Rotavirus | 0.81 (0.62, 1.04) |
| Sapovirus | 0.86 (0.74, 1.00) |
| *Shigella* | 1.02 (0.85, 1.22) |
| typical enteropathogenic *Escherichia coli* (tEPEC) | 1.20 (1.04, 1.39) |
| heat stable enterotoxigenic *Escherichia coli* (ST-ETEC) | 0.93 (0.80, 1.08) |
| *Giardia* | 0.77 (0.66, 0.89) |
| *Enterocytozoon bieneusi* | 0.70 (0.55, 0.89) |

**eFigure 2.** Prevalence of *Clostridioides difficile* detection in surveillance stools over months of the year among 1715 children enrolled in the MAL-ED birth cohort. * Denotes sites that had statistically significant seasonality (p < 0.05). Dhaka, Bangladesh, p=0.003. Naushero Feroze, Pakistan, p=0.005.


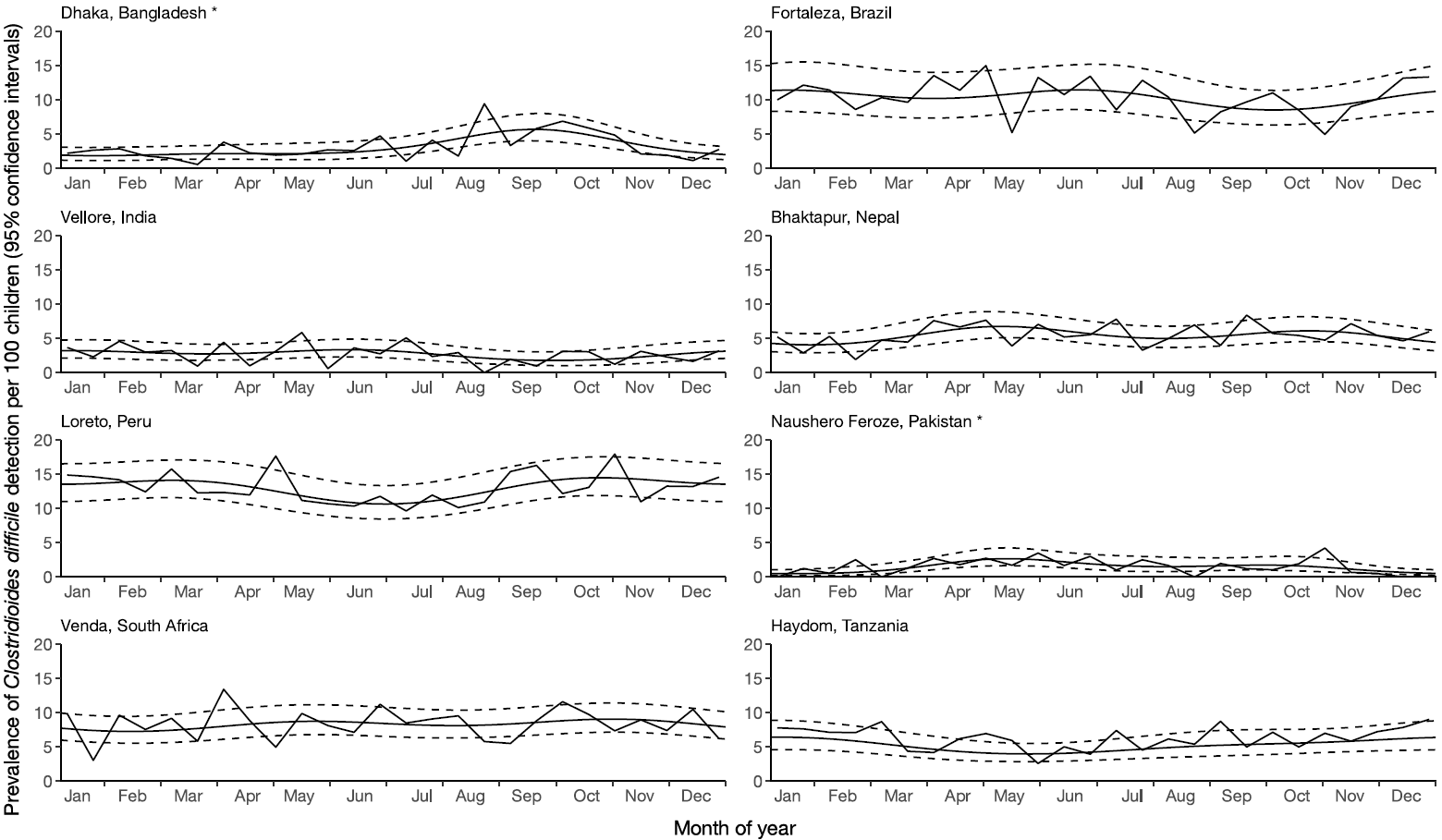


**eTable 3.** Effects of *Clostridioides difficile* burden^a^ in monthly surveillance stool samples on weight and length attainment at 2 years of age among 1469 children enrolled in the MAL-ED birth cohort

| **Site** | WAZ Difference^b,c^  β (95% CI) | LAZ Difference^b,d^  β (95% CI) |
| --- | --- | --- |
| Bangladesh |  |  |
| Burden < 10% | 0.29 (-0.01, 0.6) | 0.30 (0.04, 0.57) |
| Burden ≥ 10% | -0.16 (-0.66, 0.33) | -0.04 (-0.47, 0.39) |
| Brazil |  |  |
| Burden < 10% | 0.37 (-0.16, 0.89) | -0.04 (-0.50, 0.42) |
| Burden ≥ 10% | 0.39 (-0.09, 0.86) | 0.21 (-0.21, 0.63) |
| India |  |  |
| Burden < 10% | -0.08 (-0.35, 0.18) | -0.12 (-0.37, 0.14) |
| Burden ≥ 10% | -0.26 (-0.66, 0.13) | -0.28 (-0.66, 0.10) |
| Nepal |  |  |
| Burden < 10% | 0.01 (-0.25, 0.26) | 0.02 (-0.24, 0.28) |
| Burden ≥ 10% | -0.07 (-0.35, 0.22) | -0.08 (-0.36, 0.21) |
| Peru |  |  |
| Burden < 10% | -0.03 (-0.4, 0.35) | 0.13 (-0.23, 0.48) |
| Burden ≥ 10% | -0.17 (-0.49, 0.16) | -0.02 (-0.33, 0.29) |
| South Africa |  |  |
| Burden < 10% | -0.01 (-0.36, 0.34) | 0.16 (-0.18, 0.51) |
| Burden ≥ 10% | 0.01 (-0.35, 0.37) | 0.11 (-0.26, 0.47) |
| Tanzania |  |  |
| Burden < 10% | 0.35 (-0.02, 0.72) | 0.31 (-0.06, 0.69) |
| Burden ≥ 10% | -0.17 (-0.59, 0.24) | -0.11 (-0.53, 0.32) |
| All^e^ |  |  |
| Burden < 10% | 0.09 (-0.03, 0.21) | 0.11 (0.00, 0.23) |
| Burden ≥ 10% | -0.04 (-0.18, 0.10) | 0.01 (-0.12, 0.14) |
| Referent Group is 0 burden. LAZ = length for age z score. WAMI = Water, Assets, Maternal Education. Income, WAZ = weight for age z score. ^a^ Proportion of stool samples positive for *C. difficile* from 0-23 months of age*.* ^b^ Adjusted for sex, WAMI score, maternal height, proportion of days breastfed in the first 6 months of life, proportion of stools with *Campylobacter* detected, proportion of stools with *Giardia* detected, proportion of stools with EAEC detected, and proportion of stools with *Shigella* detected. ^c^ Adjusted for enrolment WAZ. ^d^ Adjusted for enrolment LAZ. ^e^ Adjusted for site. | | |
